# EFFECTS OF MINORITY AND CAREGIVING STRESS ON THE HEALTH OF BI+ INFORMAL DEMENTIA CAREGIVERS

**DOI:** 10.1093/geroni/igae098.0589

**Published:** 2024-12-31

**Authors:** Krystal Kittle, Ethan Cicero, Jordan Pelkmans, Jason Flatt, Joel Anderson

**Affiliations:** University of Massachusetts Amherst, Springfield, Massachusetts, United States; Emory University, Quincy, Massachusetts, United States; Emory University, Atlanta, Georgia, United States; University of Nevada Las Vegas, Las Vegas, Nevada, United States; University of Tennessee-Knoxville, Knoxville, Tennessee, United States

## Abstract

Bi+ people (e.g., bisexual, pansexual) are more likely to experience poor physical and mental health compared with monosexual minority (i.e., gay[G], lesbian[L]) people. These poor health outcomes stem from minority stress related to sexual minority status. Due to minority and caregiver stress, LGB+ people providing care to someone with Alzheimer’s disease and related dementias (ADRD) are more likely to have worse health than their straight counterparts. Little is known about how minority and caregiver stress affect the health of bi+ ADRD caregivers versus monosexual minority ADRD caregivers. Data from ADRD caregivers (bi+, n=125; gay/lesbian, n=161) were used to examine associations between minority stress, caregiver stress and overall health and if associations differed among bi+ and gay/lesbian ADRD caregivers. Multivariable regression, adjusting for demographics, was used to investigate if sexual orientation moderated the association between minority/caregiver stress variables and overall health. All minority stressors and two caregiving stressors (caring for spouse/care recipient’s level of independence) were associated with health. In our adjusted model, lifetime victimization, day-to-day discrimination, and caring for spouse/partner, moderated the association between sexual orientation and overall health. As lifetime victimization scores increased, bi+ health decreased while monosexual minority health increased. Caring for a spouse/partner, protective for both groups, had a greater effect on health for monosexual minorities. Higher day-to-day discrimination scores predicted worse health for both groups, but the magnitude was greater for monosexual minorities. Qualitative inquiries can illuminate underlying mechanisms for this phenomenon, informing interventions and policy aimed at supporting health and well-being of bi+ ADRD caregivers.

